# The Top Five Advances in Solid Organ Transplantation in the Past Fifty Years

**DOI:** 10.1002/wjs.70380

**Published:** 2026-04-30

**Authors:** Stephen J. Wigmore, Diana A. Wu

**Affiliations:** ^1^ University of Edinburgh Edinburgh Transplant Centre Royal Infirmary of Edinburgh Edinburgh UK

## 1 Introduction

The history of organ transplantation is very brief compared with the history of surgery. The 1950s and 1960s were notable for the first successful transplants for kidney, combined kidney pancreas, liver, lung and heart transplantation. These were all notable milestones which illustrated the art of the possibility but success was constrained by the rudimentary treatments available to prevent immunological organ rejection and the inability to preserve organs for more than a brief period outside of the body. At the birth of organ transplantation, immunosuppression relied on azathioprine and steroids with protocols involving whole body irradiation and or prolonged isolation. The risk of infection, cancer and bone weakness from some of these early protocols was a very significant burden for recipients. This brings us to the first of our significant advancements in organ transplantation.

## 2 Modern Immunosuppression

The introduction of ciclosporin A in 1978, a drug derived from fungi and shown to have marked immunosuppressive effects on T cells through calcineurin inhibition, transformed the immunosuppression protocols for transplant recipients [1, 2]. For the first time it became possible to reduce the dose of steroids and even withdraw them completely which avoided the steroid facies, osteoporosis and risk of infection that accompanied heavy prolonged steroid administration. Outcomes from graft rejection dramatically improved [3, 4, 5] and the later introduction of tacrolimus in 1989 [6], a similar but more potent calcineurin inhibitor with a different intracellular binding protein, resulted in further significant improvements in graft outcomes [7, 8]. Transplant clinicians soon recognized that combining immunosuppressive drugs allowed lower doses of individual drugs to be used in order to minimize toxcitiy. Mycophenolate mofetil was introduced as an alternative to azathioprine, and sirolimus as a strategy to minimize calcineurin inhibitors. Several studies have investigated steroid‐sparing strategies including antibody‐based induction alternatives [9] and early steroid withdrawal in order minimize side effects [10]. In the current era, the focus is now on the development of novel therapeutics that provide effective immunosuppression whilst minimizing the lifetime risk of infection, nephrotoxicity, metabolic complications and malignancy.

## 3 Organ Perfusion Solutions

It has been recognized since the dawn of solid organ transplantation that cessation of the circulation and removal of the organ from the body lead to a succession of cellular and physiological changes which ultimately compromise the function of the cells and the effectiveness of the organ. Cessation of the circulation effectively stops the provision of nutrients to support cellular respiration and results in the loss of ATP‐dependent proton channel pump mechanisms, leading to cellular swelling through the osmotic exchange of ions across the cell membrane. Cooling organs was recognized as a mechanism to reduce the respiratory rate within cells and potentially preserve ATP, however, cooling alone is insufficient to prevent cellular deterioration. University of Wisconsin solution or Belzer UW solution was a major leap forward in organ preservation as it was formulated to specifically counteract the biochemical and cellular injuries that occur during cold storage of organs. It was deliberately hyperosmolar containing lactobionate and hydroxyethyl starch to reduce cell swelling and had a high potassium content to limit potassium loss from cells. It also contained glutathione to preserve the molecular machinery for ATP replenishment and membrane preservation. UW solution was rich in adenosine to provide cells with the substrate for ATP regeneration on reperfusion [11]. By addressing these mechanisms of ischemic injury at a cellular level, UW solution markedly increased the time that organs could be safely preserved in cold storage whilst remaining viable with excellent outcomes. This transformed the delivery of transplant programmes enabling national and international organ sharing, improved matching and better equity for patients. One of the advantages of UW solution was that it could be used for all organs and demonstrated excellent performance for liver, kidney and pancreas [12]. Other solutions such as HTK solution for kidneys and Stein solution for lungs achieved popularity but many of these distinct solutions borrow on the science behind UW solution.

## 4 Expanded Indications and Types of Transplantation

The great majority of solid organ transplants are undertaken to save life in organ failure. Increased safety of immunosuppression and extended organ survival in recipients allowed the development of transplantation for other indications. One of the most widely accepted non‐liver failure indications is liver cancer. Hepatocellular carcinoma (HCC) in particular has excellent outcomes with liver transplantation often surpassing outcomes achievable with resection or locoregional therapy. Various criteria have been devised to select the patients most likely to benefit from liver transplantation for HCC. Programmes continue to explore the role of liver transplantation for non‐HCC cancers such as metastatic neuroendocrine and colon cancer, epithelioid hemangioendothelioma and cholangiocarcinoma.

In terms of expanding types of transplantation, one of the most headline‐grabbing innovations was face transplantation. This procedure was first successfully undertaken in Paris in 2005 for a woman who had suffered severe facial injuries from being attacked by a dog [13]. Extensive discussion around ethics of face transplantation followed, with much of the media debating the science fiction and potential crime misuse. In doing so, the real motives for face transplantation were not fully acknowledged, which were to restore function in people with severe burns or other facial injuries that were threatened with losing sight because of lack of eyelid function, were unable to eat, drink or speak normally because of tissue loss around the lips and mouth and who had unreconstructible disfigurements that made them fear societal rejection. Face transplantation was followed shortly after by hand and arm transplantation [14, 15]. The complexity of these mixed tissue composite allografts transplants is truly amazing requiring multiple surgeons working over many hours to join nerves, arteries, veins, bones, tendons and even lymphatics to restore function to individuals with limb loss. Both face and limb transplantation recipients require extensive psychological counseling and one of the complications is rejection of the donor limb by the recipient who cannot comprehend it as their own. With the development of functional transplantation, the question arose as to whether it would be possible to transplant a uterus into an individual born without one, to address this awful barrier to fertility. Uterus transplantation can now be performed either as a cadaveric or living donor procedure with the implanted uterus capable of supporting normal growth and development of a fetus [16, 17]. Since a uterus transplant is an allograft, it is necessary for the recipient to take immunosuppression during pregnancy. However, as this is another example of a transplant restoring function, it is possible to withdraw immunosuppression after delivery and remove the transplanted uterus. In this way the woman receiving the uterus transplant does not need to face the long‐term consequences and risks of immunosuppression.

## 5 Ex Vivo Organ Assessment and Support

The drive to extend the life of organs has led to the development of organ perfusion machines, in the hope that this might improve transplant outcomes further. The first machines were little more than pumps driving cold perfusion fluid through the organ. These were particularly used in renal transplantation and the data from large randomized controlled trials of kidney perfusion versus static cold storage showed improved outcome in terms of reduced rates of delayed graft function and better graft survival [18]. Anecdotally the mechanical perfusion of kidneys often also improved the graft appearance due to the removal of microthrombi in the renal vasculature which gave rise to an unappealing blotchy appearance. Liver perfusion devices followed the same pattern of development with cold perfusion of livers first performed before the introduction of normothermic machine perfusion (NMP). A number of NMP systems have been developed but the leader in terms of evidence is the Organox Metra device. A randomized controlled trial of donor livers allocated to NMP or static cold storage showed improved organ utilization and reduced evidence of ischemia reperfusion injury [19]. Interestingly, normothermic machine perfusion has not been able to prevent the ischemic cholangiopathy associated with donation after cardiac death liver transplantation. Another mechanical perfusion approach to the donor is normothermic regional perfusion, which involves the use of a circuit similar to extracorporeal machine oxygenation (ECMO) to perfuse organs in situ in the deceased donor. This technology has demonstrated improvements in graft outcome for liver, kidney and pancreas transplant and has been able to reduce the rate of ischemic cholangiopathy [20, 21, 22]. Similar advancements have been made with machine perfusion of hearts and lungs and the “heart in a box” and “lung in a box” devices are improving outcomes particularly for donation after cardiac death thoracic transplants. Machine perfusion offers additional advantages beyond improving graft outcomes. In particular, in liver, heart and lung transplantation the ability to test an organ while on pump is allowing the development of criteria to differentiate organs likely to succeed from those with poor predicted function. The next step is drug or cell therapy on pump, to improve organ performance. Machine perfusion has also allowed the timing of transplantation to change, extending the time that an organ can be implanted, so that complex procedures can take place in daylight hours rather than in the middle of the night.

## 6 Living Donor Transplantation Technique Refinement

In a significant number of countries around the world religious or cultural beliefs or the lack of a robust legal framework around the diagnosis of death have prevented the development of cadaveric transplantation. The development of living donor transplantation has made solid organ transplantation accessible to populations that would have had no such opportunity ordinarily. Ironically the first successful kidney transplants were undertaken as living donor operations often between consanguineous twins. Having two kidneys and more renal function than is necessary for homeostasis allows kidney donation and careful selection minimizes the risk to the donor. Kidney donation has become popular as an altruistic act and altruistic donors can be used to initiate chains of incompatible donor ‐recipient pairs, who each donate a kidney to another unrelated person so that their friend or relative can receive a compatible kidney transplant themselves. Living liver donation is a more challenging prospect since partial liver donation involves division of the liver parenchyma but must obviously preserve sufficient volume and function for the donor as well as suitable volume and function for the recipient. Techniques in liver splitting and vascular reconstruction have allowed adult to child liver transplantation including segmental and subsegmental transplants in neonates up to full adult to adult liver transplantation with right lobe grafts, full left lobe grafts and even two left lateral sections (segments II & III) from two separate donors [23, 24]. The reasons for the multiplicity of living liver donor procedures reflects the size differences between adults and children (including babies) as well as a desire to reduce the morbidity and risk to healthy living liver donors. Minimally invasive kidney, liver and lung donation procedures have all been described and represent a significant advance in reducing morbidity for the donor. Living lung donation is now also possible.

There have been so many notable advances in transplantation in the past 50 years that it has been difficult to limit these to just five. Notable mentions should also be made of the clinical impact of improved understanding of the human leukocyte antigen (HLA) system and its importance in immune rejection and the ability of tissue type matching to obviate some rejection scenarios or improve outcomes for patients on standard immunosuppression. Similarly, none of the successes in transplantation would have been possible without the ethical and legal frameworks that administrations put in place to regulate such a sensitive and important branch of medicine. Possibly because solid organ transplantation was often seen as an experimental therapy, it has been subject to some of the most robust auditing and regulation. This has allowed the creation of high‐quality databases of transplant outcomes and performance and has been a rich foundation for scientific development in the field. What is absolutely clear is that solid organ transplantation is still advancing. The potential of multiply gene‐edited xenotransplants, removing the genes encoding animal antigens, porcine retroviruses and other barriers to use in humans is only just becoming realized. Similarly, the use of cell therapy to repair organs in situ or to be used for transplantation offers huge potential for obviating the need for transplant or expanding the organ donor pool.

## Author Contributions

**Stephen J. Wigmore:** conceptualization, writing – original draft, writing – review and editing, validation, project administration, supervision. **Diana A. Wu:** conceptualization, writing – original draft, writing – review and editing, project administration.

## Funding

The authors have nothing to report.

## Conflicts of Interest

The authors declare no conflicts of interest.

## Data Availability

Data sharing not applicable to this article as no datasets were generated or analysed during the current study.
